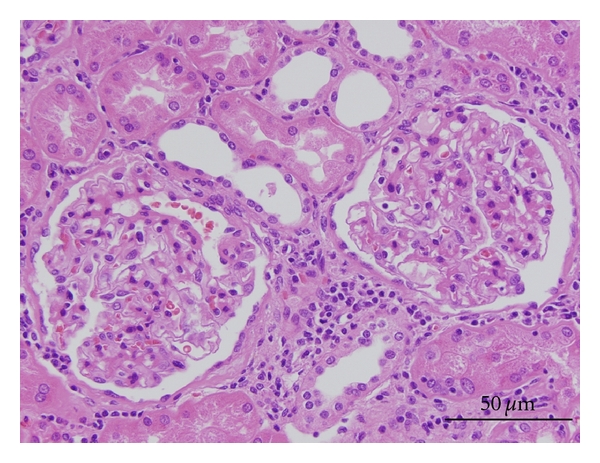# Development of Nephrotic Syndrome after Administration of Sorafenib in a Case of Metastatic Renal Cell Carcinoma

**DOI:** 10.1155/2011/710216

**Published:** 2011-10-05

**Authors:** Yumiko Okuno, Haruki Kume, Chihiro Hosoda, Yukio Homma

**Affiliations:** Department of Urology, The University of Tokyo Hospital, Tokyo 113-8655, Japan

## Abstract

Nephrotic syndrome, after administration of tyrosine kinase inhibitors, is uncommon and not well known. A 62-year-old male, who had experienced a left nephrectomy due to a traffic accident 38 years ago, underwent a partial nephrectomy for right renal cell carcinoma (RCC). Histologically, the tumor was a clear cell RCC. Two years later abdominal CT revealed para-aortic lymph node metastasis. During these two years, serum creatinine had increased from 2.0 mg/dL to 2.9 mg/dL along with the appearance of proteinuria. After only a week of sorafenib, 400 mg/day, fever developed and sorafenib was stopped. Although normotensive, his serum creatinine increased to 3.83 mg/dL and serum albumin decreased from 1.8 g/L to 1.0 g/L. Proteinuria also worsened to 27.5 g/day. He became edematous, and ascites and cardiac effusions also appeared. He was diagnosed with nephrotic syndrome. A retrospective review of the histology of the partial nephrectomy revealed no change in the glomeruli.

## 1. Introduction

Recently, vascular endothelial growth factor (VEGF) receptor blockers have been commonly used for treating advanced renal cell carcinoma (RCC). Although renal dysfunction caused by these inhibitors is uncommon, it is important for advanced RCC because many RCC cases have chronic kidney disease [1, 2]. However, this uncommon adverse effect has not been well documented.

## 2. Case Report

A 62-year-old male, who had been subjected to a left nephrectomy due to a traffic accident at the age of 24, was admitted for treatment of a right renal cell carcinoma, which measured 11 cm in diameter. Partial nephrectomy was performed that included 25 minutes of warm ischemic time. Histologically, the tumor was a clear cell RCC without perinephric fat or microvascular invasions.

Two years after the surgery, abdominal CT revealed para-aortic lymph node metastasis, 2.5 cm in diameter, which was confirmed histologically by CT-guided biopsy. During the two years after the partial nephrectomy, serum creatinine had increased gradually from 2.0 mg/dL to 2.9 mg/dL together with the appearance of proteinuria.

Sorafenib, 400 mg/day, was started but after only a week of administration a fever developed and sorafenib was stopped. Although he was normotensive after the cessation of sorafenib, serum creatinine increased to 3.83 mg/dL and serum albumin decreased from 1.8 g/L to 1.0 g/L. Proteinuria also worsened; the amount of urinary protein was 27.5 g/day. He became edematous and his body weight increased from 53.5 kg to 57.6 kg. Ascites and cardiac effusions also appeared.

He was diagnosed with nephrotic syndrome. With sodium restriction, supplementation of albumin, and administration of diuretics, his edema, ascites, and cardiac effusions were improved. The amount of urinary protein was also reduced to 9.4 g/day.

Renal biopsy was not performed because of the possible complications that could progress to deterioration of renal function. A retrospective review of the histology of the partial nephrectomy revealed no change in the glomeruli (Figure 1).

## 3. Discussion

Ablation of the kidney results in proteinuria, hypertension, progressive renal failure, and advanced glomerulosclerosis in animal models [1]. Also, clinically in chronic kidney disease, glomerular filtration rate under 60 mL/min is known to develop within three years in two thirds of cases that underwent radical nephrectomy [2]. As this case involved a partial nephrectomy for RCC in a solitary kidney, only a small volume of parenchyma remained, which could cause progression of renal dysfunction after the surgery.

Nephrotic syndrome induced by tyrosine kinase inhibitors (TKIs) such as sorafenib and sunitinib has been reported only in several cases [3–9], although it seems more common in cases treated by bevacizumab, a humanized monoclonal antibody neutralizing VEGF. However, the etiology is not fully understood [10]. VEGF, produced by podocytes, activates VEGF receptor 2 on glomerular capillary endothelial cells. Its inhibition may cause a loss of endothelial fenestrations and reduced proliferation of endothelial cells. Thrombotic microangiopathy and hypertension induced by anti-VEGF therapy may play a role [10].

Before administration of sorafenib, this case was already in renal insufficiency with low serum albumin and proteinuria, although it did not meet the diagnostic criteria of nephrotic syndrome. However, cases without proteinuria have been reported to develop nephrotic syndrome after TKI treatment [3–7]. Although the risk factors for renal dysfunction after anti-VEGF therapy remain unknown, this infrequent adverse effect cannot be ignored.

## Figures and Tables

**Figure 1:**